# Interaction of RNA viruses of the natural virome with the African malaria vector, *Anopheles coluzzii*

**DOI:** 10.1038/s41598-019-42825-3

**Published:** 2019-04-19

**Authors:** Ferdinand Nanfack-Minkeu, Christian Mitri, Emmanuel Bischoff, Eugeni Belda, Isabelle Casademont, Kenneth D. Vernick

**Affiliations:** 1 Department of Parasites and Insect Vectors, Unit of Genetics and Genomics of Insect Vectors, Institut Pasteur, Paris, 75724 CEDEX 15 France; 2 CNRS, Unit of Evolutionary Genomics, Modeling and Health (UMR2000), 28 rue du Docteur Roux, 75015 Paris, France; 3 Sorbonne Université, Graduate School of Life Sciences ED515, UPMC - Université Pierre et Marie Curie - Paris 6, 4 Place Jussieu, 75252 Paris, France; 4 Unit of Functional Genetics of Infectious Diseases, Department Genome and Genetics, Paris, France

**Keywords:** Virus-host interactions, Entomology

## Abstract

Mosquitoes are colonized by a little-studied natural virome. Like the bacterial microbiome, the virome also probably influences the biology and immunity of mosquito vector populations, but tractable experimental models are lacking. We recently discovered two novel viruses in the virome of wild *Anopheles* and in colonies of the malaria vector *Anopheles coluzzii*: *Anopheles* C virus and *Anopheles* cypovirus. Here, we describe biological interactions between these two viruses and *An*. *coluzzii* mosquitoes. Viral abundance varies reproducibly during mosquito development. DNA forms of these viruses were not detected, and thus viral persistence is likely based on vertical transmission of RNA genomes. At least *Anopheles* C virus is vertically transmitted by an intraembryonic route. Relative abundance of the two viruses is inversely correlated in individual mosquitoes. One possible mechanism for this could be interactions with host immunity, and functional genomic analysis indicated differential influence of at least the Toll and JAK/STAT immune signaling pathways upon the viruses. The nonrandom distributions and interactions with host immunity suggest that these and other members of the natural virome may constitute a source of unrecognized heterogeneity in mosquito vector populations.

## Introduction

*Anopheles* species are the main vectors of human malaria. In addition to *Plasmodium*, *Anopheles* mosquitoes transmit filarial worms and arboviruses^1,2^. *Anopheles* mosquitoes also harbor a diverse natural virome of RNA viruses^2–5^. The *Anopheles* virome is composed mainly of insect specific viruses (ISVs) that multiply only in insects, but also includes relatives of arboviruses that can replicate in both insects and vertebrates. However, *Anopheles*-RNA virus interactions have been relatively unexamined, and it is unknown why *Anopheles* are efficient vectors of human malaria but do not transmit viruses as well as *Aedes* and *Culex* mosquitoes.

The only arbovirus known to be consistently transmitted by *Anopheles* mosquitoes is O’nyong nyong virus (ONNV, genus *Alphavirus*, family *Togaviridae*). ONNV is mainly transmitted by *An*. *gambiae* and *An*. *funestus*, two major African malaria vectors^6^. Millions of people were infected in known ONNV epidemics^6–8^. *Anopheles* species may also contribute to transmission of other arboviruses such as West Nile virus (WNV), Japanese encephalitis virus, and Wesselsbron virus^2,5,9,10^.

*Anopheles* antiviral immunity to ONNV has been studied^11–13^, but the mechanisms of competence and immunity of these mosquitoes to other RNA viruses are essentially unknown. RNA interference (RNAi), Toll, Imd and JAK/STAT are the main antiviral immune signaling pathways described in mosquitoes. The RNA interference (RNAi) pathway controls ONNV replication in *An*. *coluzzii* during the disseminated systemic infection^11,12,14^, but RNAi has no detectable protective function against the primary blood-induced infection of the midgut epithelium^12^. Conversely, the activity of the JAK/STAT and Imd pathways are required for protection against the primary midgut infection by ONNV, but play no protective role against the disseminated infection^12^. Thus, distinct antiviral mechanisms function against the primary midgut infection or the disseminated systemic infection.

However, besides ONNV, no studies have been reported on the functional interactions between *Anopheles* mosquitoes and other RNA viruses, to our knowledge. Studies of other RNA viruses in *Anopheles* would indicate the specific or general nature of the antiviral mechanisms observed for ONNV. Deep sequencing has recently facilitated discovery of replicating RNA viruses in insects, by identifying viral RNA (viRNA) products of siRNA cleavage of the double-stranded RNA intermediates of active viral replication^15,16^. By this approach, we recently identified two viruses in *An*. *coluzzii*: *Anopheles* C virus (AnCV) and *Anopheles* cypovirus (AnCPV)^16^. Wild *Anopheles* of diverse species collected in Senegal and Cambodia were positive for AnCPV, and AnCV was present in samples from Senegal. Therefore, both viruses belong to the natural virome of *Anopheles*.

A recent survey found published evidence of at least 51 viruses naturally associated with *Anopheles*^5^. Because *Anopheles* are persistently exposed in nature to members of the natural virome, it is likely that these ISVs have been the main evolutionary pressure shaping *Anopheles* antiviral immunity, rather than the less frequent exposure to arboviruses such as ONNV. Thus, here we characterize the biology and host in the interaction of *Anopheles* with two RNA viruses from the natural virome, AnCPV and AnCV. Understanding the biology underlying the apparent inefficiency of arbovirus transmission by *Anopheles* may reveal potential new tools to control virus transmission efficient arbovirus vectors such as *Aedes* and *Culex*.

## Results

### Relative composition of the virome varies during mosquito development

**Figure 1:**
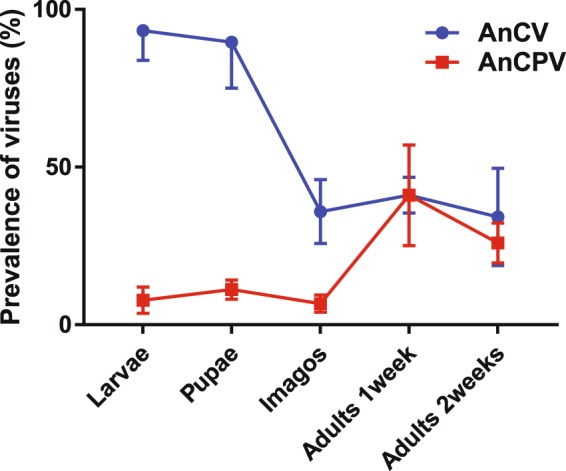
Infection prevalence of viruses varies reproducibly during development of *Anopheles coluzzii*. Virus levels were measured by RT-PCR. Each line represents average infection prevalence for *Anopheles* C virus (AnCV, circles) and *Anopheles* cypovirus (AnCPV, squares). Error bars indicate standard deviation from three biological replicates (25–30 individuals per replicate) of each *Anopheles* stage: larvae (n = 90), pupae (n = 88), imagos (n = 89), adults 1 week (n = 90), adults 2 weeks (n = 88). Detailed descriptive statistics in Supplementary Table S1.

It was previously shown that *Anopheles* C virus (AnCV) and *Anopheles* cypovirus (AnCPV) are present in the natural virome of wild *Anopheles*, and also in *Anopheles* laboratory colonies, including all colonies of *An*. *coluzzii* tested to date^16^. Despite the presence of at least 51 viruses detected in *Anopheles* species worldwide^5^, AnCV and AnCPV were the only RNA viruses detected in the Ngousso colony of *An*. *coluzzii*^16^, and the biology of these two viruses is the topic of the current study. To determine the abundance of the two viruses during host development, we measured their prevalence by RT-PCR in different life stages of the *An*. *coluzzii* Ngousso strain (Fig. 1, Supplementary Table S1). Infection prevalence of AnCV decreases between pupae and newly emerged adults (p = 2.79e-07, chi-square = 41.08, df = 6, 3 biological replicates), suggesting an impact of metamorphosis on AnCV replication. In contrast, infection prevalence did not change between larvae and pupae, or between 1 week and 2-week-old adults for either virus. The infection prevalence of the two viruses displays a consistent reciprocal pattern in the aquatic larval and pupal stages, with high prevalence of AnCV, while AnCPV infection prevalence is low in larval stages and newly emerged adults, increasing in adults by 1 week after emergence.

### Absence of DNA forms for both AnCV and AnCPV

DNA forms of non-retroviruses have been implicated in viral persistence in *Drosophila* and *Aedes* species^17,18^. We surveyed *An*. *coluzzii* to detect DNA forms of AnCV and AnCPV by PCR using three specific primer sets directed against each virus. The template was genomic DNA extracted from *An*. *coluzzii* pools, or cDNA generated from RNA of mosquito pools. Despite the high infection prevalence of these viruses, no specific amplification was detected from DNA template (Supplementary Fig. S1). As a positive control, PCR amplification was detected in all cDNA pools, and the larger size of the control ribosomal protein S7 (rpS7) product in the DNA reactions, due to the unspliced intron, confirmed the DNA source of the template. To further strengthen this observation, both virus sequences were used to query the *An*. *gambiae* PEST genome assembly using blastn, and no significant matches were detected. Taken together these results indicate that persistence of AnCV and AnCPV in *An*. *coluzzii* is probably not based on transmission of DNA forms of the virus genomes.

### Transovarial intraembryonic route of AnCV persistence

**Figure 2:**
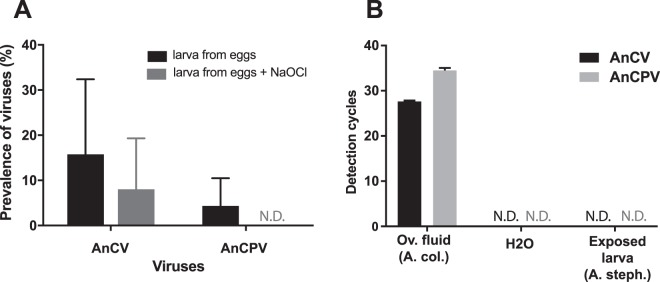
Vertical transmission of Anopheles C virus is intraembryonic. (**A**) Evaluation of transovarial and transovum routes of AnCV and AnCPV vertical transmission using an RT-PCR to detect both viral genomes in *An*. *coluzzii* larvae hatched from eggs in individual wells. Control larvae (black bars) were hatched from eggs mock-treated without NaOCl, and carry both viruses. Larvae grown from eggs treated with NaOCl (gray bars) carry AnCV, while AnCPV was not detected after treatment (N.D.). (**B**) Both viruses were present in oviposition fluid washed from the surface of *An*. *coluzzii* eggs (Ov. Fluid (A. col.)). Viruses were not detected in the pre-rinse water (H2O, N.D.). The virus-positive oviposition fluid was fed to uninfected *An*. *stephensi* in larval water, and the exposed larvae did not become detectably infected (Exposed larva (A. steph.), N.D.). Experiments were done in two replicates, error bars indicate standard deviation.

It was previously observed that AnCV and AnCPV are vertically transmitted in *An*. *coluzzii* colonies^16^, but the transmission routes were not determined. Viruses can pass to progeny within the eggs by a transovarial intraembryonic route, or by egg surface contamination by a transovum route, for example in ovarian fluid. To distinguish between these routes, *An*. *coluzzii* eggs were treated with 0.025% sodium hypochlorite (NaOCl) to inactivate viruses on the egg surface, rinsed, and eggs were placed individually in a well of 24-well plates for hatching to prevent horizontal virus transmission between larvae. Controls were treated identically, but without exposure to NaOCl. Larvae (L3/L4 stages) were tested by RT-PCR. Only larvae could be tested because individual eggs reared to larvae in individual wells of a plate did not efficiently develop to the adult stage. Larvae from untreated eggs were infected with both viruses, and larvae from the NaOCl-treated group were infected with detectable levels of at least AnCV (Fig. 2A).

Liquid rinsed from the surface of *An*. *coluzzii* eggs containing oviposition fluid was tested using duplex Taqman real-time RT-PCR (Taqman RT-qPCR), to simultaneously measure abundance of both viruses. The oviposition fluid washed from eggs was orally administered to *An*. *stephensi* larvae by feeding in the water, to test for virus particles competent for horizontal transmission. The oviposition fluid from *An*. *coluzzii* was positive for RNA of AnCV and AnCPV, however the exposed *An*. *stephensi* larvae were not infected by either of the two viruses (Fig. 2B). As a positive control, we previously showed that exposure of *An*. *stephensi* larvae by the same methodology to filtered extract of homogenized *An*. *coluzzii* Ngousso larvae caused infection of 100% of exposed *An*. *stephensi* larvae^16^. Although we cannot rule out a dose effect of virus load difference between oviposition fluid and larval extract, this result may also suggest that viruses in oviposition fluid on the egg surface could be less infectious or non-infectious as virus particles. Taken together, these results suggest that vertical transmission is unlikely to be maintained by simple environmental contamination of oviposition sites with maternal oviposition fluid. These data indicate that an intraembryonic transovarial route is involved in the vertical transmission of at least AnCV, and consistent maintenance by an egg surface transovum route does not seem likely for either virus.

### Abundances of AnCV and AnCPV are inversely correlated in mosquitoes

Host interaction with the virome establishes the basal physiological and immune environment encountered by arboviruses, which are probably not frequent components of the pre-existing virome. Thus, interactions among members of the virome might have implications for susceptibility of the host to arboviruses. For example, infection with Nhumirim virus, an insect-specific flavivirus found in the insect virome, reduced by 10,000-fold the viral load of West Nile virus in *Ae*. *albopictus* cells, and may serve as a barrier to WNV transmission by *Culex*^19^. Cases of homologous and heterologous viral interference were described with mosquito cells^20^. Knowledge of viral interactions is lacking in *Anopheles*.

**Figure 3:**
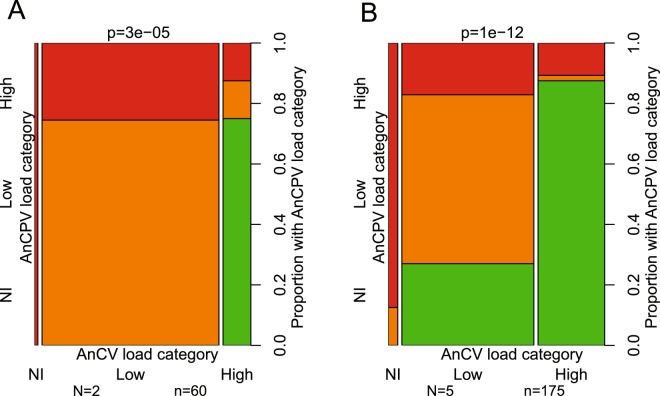
Inverse correlation between AnCV and AnCPV infection levels in *Anopheles coluzzii*. Spine plots depict the conditional probability of AnCPV infection load category given AnCV infection load category as measured by duplex Taqman RT-qPCR. The right y-axis indicates the proportion of mosquitoes with the given AnCPV load category (left y-axis) within each AnCV load category (x-axis). Mosquito viral loads were categorized as Non-infected (NI, green), Low infected (orange), and High infected (red) for each virus. Infection levels were defined as virus signal relative to *An*. *coluzzii* ribosomal protein rpS7 control, where Low and High levels were values smaller or greater than log10 = 0, respectively, and Non-infected displayed absence of specific virus PCR signal. Infection category of AnCV is indicated by the three histogram bars on the x-axis (AnCV load category), and AnCPV by the three bars on the left y-axis (AnCPV load category). Bar widths on the x-axis are proportional to the total number of mosquitoes in each AnCV load category. (**A**) Virus infection levels measured in untreated mosquitoes. (**B**) Virus infection levels measured in mosquitoes wounded by injection with control double stranded RNA, dsGFP. (N) indicates the number of biological replicates and (n) the total number of mosquitoes for all replicates. Statistical difference based on a null hypothesis of random distribution of the two viruses was first tested independently within replicates by the chi-square test using 100,000 permutations to assess the p-value. Individual replicates displayed a consistent direction of change (that is, inverse correlation of abundance of the two viruses, Supplementary Fig. S2), and consequently individual p-values were combined using the method of Fisher to generate a combined p-value, shown above each plot. Detailed statistics in Supplementary Table S4.

*An*. *coluzzii* is persistently coinfected with both AnCV and AnCPV, and we examined the relationship between abundance of the two viruses in individual mosquitoes. Infection intensity and prevalence of both viruses was measured simultaneously in RNA of individual mosquitoes using duplex Taqman RT-qPCR. The abundance of the two viruses in individual mosquitoes is not independent, but rather is inversely correlated. Mosquitoes with high AnCV infection intensity displayed significantly lower infection intensity with AnCPV, and the reciprocal (Fig. 3A; combined p = 3e-05, chi-square = 26.81, df = 4; replicates shown in Supplementary Fig. S2). Co-infection occurs only when both viruses are present at low viral loads.

Physical injury of *Anopheles* triggers expression of a suite of wound-response genes that render mosquitoes more resistant to *Plasmodium falciparum*^21^. Therefore, we also measured virus abundance distributions in mosquitoes that were wounded by injection of buffer with an irrelevant double-stranded RNA directed against Green Fluorescent Protein. The wounded mosquitoes displayed the same significant inverse correlation of virus abundance as non-injected mosquitoes (Fig. 3B; combined p = 1e-12, chi-square = 76, df = 10; replicates shown in Supplementary Fig. S2). The p-value is more significant from the five combined replicates of wounded mosquitoes (Fig. 3B) as compared to the two combined non-injected replicates (Fig. 3A), however p-values of the individual replicates are comparable for untreated and wounded mosquitoes, indicating that wounding does not alter the conditions that influence distributions of the two viruses. These results indicate that infection efficiency of each virus is not independent of the other, but does not shed light on the mechanism. Potential mechanisms include interference by virus manipulation of the cellular environment and/or host immunity to exclude the other virus, or segregating host genetic differences that are favorable to one virus or the other. We next examined the interaction of the two viruses with host immunity.

### The Toll signaling pathway influences AnCPV abundance in mosquitoes

Maintenance of ISVs in a non-pathogenic or commensal state probably requires active policing by basal host immunity, similar to the continual dialog between host immunity and the bacterial microbiome^22^. AnCV and AnCPV are widespread members of the natural *Anopheles* virome, and it is likely that the mosquito host deploys immunity to limit viral replication and potential pathogenesis. Here, we used RNAi-mediated gene silencing assays to query the influence of key immune signaling pathways on AnCV and AnCPV abundance.

The Toll pathway controls rodent malaria parasite infection in *Anopheles*^23,24^ and limits replication in *Aedes* of dengue (DENV, genus Flavivirus, family Flaviviridae) and Semliki forest viruses (SFV, genus Alphavirus, family Togaviridae)^25,26^. In *Anopheles*, the Toll pathway influence on viruses has only been studied for ONNV^12^. ONNV strongly inhibits activation of Toll in *Anopheles*, possibly as an adaptive mechanism because when ectopically activated, Toll significantly inhibits ONNV replication. DENV and SFV are also able to inhibit signaling of Toll in *Aedes*^27,28^.

We examined the role of the Toll pathway in the control of AnCV and AnCPV in *An*. *coluzzii* by silencing Cactus, a negative regulator of the Toll pathway, to activate Toll signaling (silencing validation shown in Supplementary Fig. S3). Mosquitoes were injected with double-stranded RNA targeting Cactus transcript (dsCactus), and virus abundance was measured by duplex Taqman RT-qPCR. Aggregate analysis of all mosquitoes after silencing Cactus displayed a non-significant tendency of lower AnCV infection intensity (Supplementary Fig. S3a) and inconsistent results for AnCPV infection intensity (Supplementary Fig. S3b).

**Figure 4:**
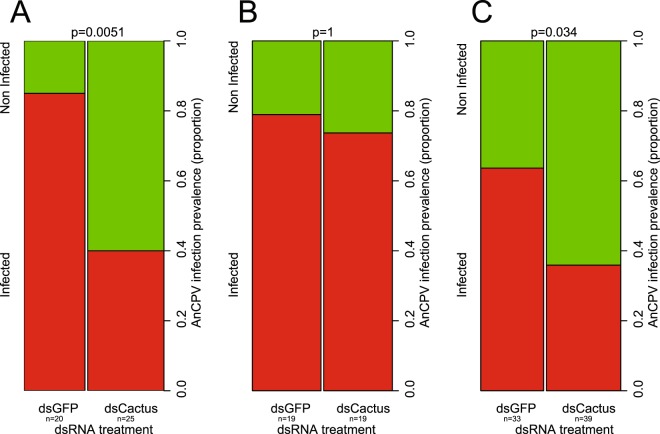
Toll pathway activity limits levels of AnCPV in *Anopheles coluzzii*. Silencing the Toll negative regulator Cactus activates Toll signaling, and decreases the prevalence of AnCPV when analyzed in the AnCV low-infected group of mosquitoes to minimize the interference effect from AnCV. AnCV low infection level was defined as in Fig. 3 as the AnCV Non-infected and Low-infected groups combined. Plots show the prevalence of infection by AnCPV as a proportion, in three biological replicates ((**A**) replicate 1, (**B**) replicate 2, (**C**) replicate 3). dsCactus indicates mosquitoes injected with double-stranded RNA directed against Toll negative regulator Cactus transcript, and dsGFP indicates mosquitoes injected with irrelevant dsRNA. X-axis, dsRNA treatment, y-axis, AnCPV infection prevalence. (n) indicates the total number of mosquitoes for each of three biological replicates. Statistical differences were first tested independently within replicates by the Wilcoxon-Mann-Whitney test using 100,000 permutations to assess the p-value (given above each replicate plot). Individual replicates displayed a consistent direction of change, and consequently individual p-values were combined using the method of Fisher to generate a combined p-value (combined p = 0.0013, chi-square = 21.8, df = 6). Detailed statistics in Supplementary Table S4.

However, considering the inverse correlation of abundance between the two viruses and potential interference effects (Fig. 3), we then analyzed the effect of Cactus silencing on AnCPV by focusing on the group with low or no AnCV load (equivalent to NI and Low infection categories in Fig. 3). This analysis revealed that Toll activation by dsCactus treatment significantly reduced AnCPV infection prevalence as compared with the dsGFP controls (Fig. 4; combined p = 0.0013, chi-square = 21.8, df = 6). The NI and Low AnCV groups were combined because NI mosquitoes alone were rare, and thus there was no power to compare AnCPV infection levels between these latter two groups. These results support the conclusion that AnCPV is sensitive to antiviral activity mediated by the Toll pathway, while AnCV is insensitive to the effects of the Toll pathway. High AnCV infection also appears to cause interference against AnCPV, which suggests the complexity of the trade-offs that structure the ecology of the virome.

### JAK/STAT signaling activity promotes AnCPV infection in *Anopheles coluzzii*

**Figure 5:**
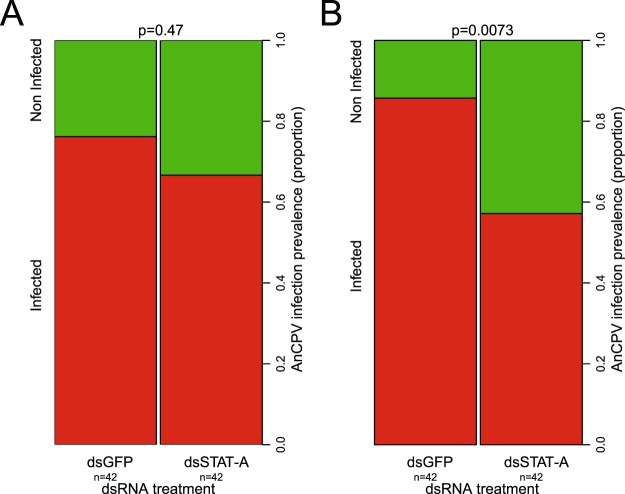
JAK/STAT pathway activity promotes AnCPV infection in *Anopheles coluzzii*. Effect of STAT-A silencing on AnCPV prevalence is indicated as a proportion in two biological replicates ((**A)** replicate 1, (**B**) replicate 2). dsSTAT-A indicates mosquitoes injected with double-stranded RNA directed against STAT-A transcript, and dsGFP indicates mosquitoes injected with irrelevant dsRNA. X-axis, dsRNA treatment, y-axis, AnCPV infection prevalence. (n) indicates the total number of mosquitoes for each of the 2 replicates. Statistical differences were first tested independently within replicates by the Wilcoxon-Mann-Whitney test using 100,000 permutations to assess the p-value (given above each plot). Individual replicates displayed a consistent direction of change, and consequently individual p-values were combined using the method of Fisher to generate a combined p-value (combined p = 0.02, chi-square = 11.4, df = 4). Detailed statistics in Supplementary Table S4.

The Janus kinase/signal transducer and activator of transcription (JAK/STAT) pathway is implicated in antiviral protection in *Anopheles* against ONNV^12^, and in *Aedes* against DENV and SFV^25,26^, as well as in mice and Drosophila^29,30^. We silenced STAT-A, a positive regulator of the JAK/STAT pathway, in *An*. *coluzzii* mosquitoes in order to inhibit activation of the pathway (silencing validation shown in Supplementary Fig. S4), and measured virus infection levels by duplex Taqman RT-qPCR. Inhibition of JAK/STAT decreased AnCPV infection prevalence of mosquitoes (Fig. 5, combined p = 0.02, chi-square = 11.4, df = 4, for 2 replicates). There was no effect on AnCPV infection intensity (replicate 1 p = 0.5, replicate 2 p = 0.29), and no effect on AnCV infection (replicate 1 p = 0.37, replicate 2 p = 0.62). This result indicates that the JAK/STAT immune pathway plays a significant role in promoting infection by this cypovirus member of the virome. Interestingly, unlike for the Toll pathway, the effect of STAT-A silencing was detected by the aggregate analysis of all mosquitoes after silencing STAT-A, and did not require analysis of only the low-AnCV fraction of mosquitoes for power to detect an effect. This suggests that, unlike for Toll, AnCV does not appear to influence activation of the JAK/STAT pathway, and/or the influence of the pathway on AnCPV replication.

### RNA interference, Imd, and Pastrel do not significantly influence AnCV and AnCPV levels in *An*. *coluzzii*

The RNA interference (RNAi) pathway is a major antiviral mechanism in Drosophila and mosquitoes^31–33^, including in *Anopheles* against ONNV^11,12,14^. Argonaute-2 (Ago2) is a key factor of the RNAi pathway, and an antagonist of ONNV in *Anopheles*. To assess the influence of the RNAi pathway for the control of AnCV and AnCPV in *An*. *coluzzii*, Ago2 was depleted by silencing, and virus levels were measured by duplex Taqman RT-qPCR (silencing efficiency shown in Supplementary Fig. S5). There was no consistent effect of inhibition of RNAi activity by Ago2 silencing upon the abundance of AnCV and AnCPV, and none of the replicates was individually significant (Supplementary Fig. S5a). Moreover, AnCPV prevalence in the group with low or no AnCV load (equivalent to NI and Low infection categories in Fig. 3) was also not significantly affected in two independent replicates (p = 1, combined Fisher test).

The immune deficiency (Imd) pathway plays an antiviral role against ONNV during the primary midgut infection of *An*. *coluzzii*^12^, but has no effect during the secondary disseminated systemic infection^14^. There are two isoforms of the Imd positive activator, Rel2: the longer Rel2-F and shorter Rel2-S. Silencing of both isoforms by targeting a shared region, and specifically silencing only Rel2-F, both caused increased ONNV infection in the midgut, and thus Imd-mediated protection against ONNV in the midgut requires at least the long Rel2-F isoform. Here, we targeted both isoforms by injecting a double-stranded RNA (dsRel2) directed against the common region (silencing efficiency by RT-qPCR shown in Supplementary Fig. S6). Depletion of Rel2 led to a consistent but non-significant tendency in three replicates towards lower infection prevalence for AnCPV (Supplementary Fig. S6a). AnCPV prevalence in the group with low or no AnCV load was not significantly affected in three independent replicates (p = 0.98, combined Fisher test).

Pastrel (pst) has been reported in Drosophila as an antiviral factor against picorna-like viruses and some dicistroviruses, such as Drosophila C-virus, a dicistrovirus related to AnCV^34–36^. There is currently no pst ortholog annotated in the *An*. *gambiae* PEST genome assembly. By searching for reciprocal best hits, we identified *An*. *gambiae* AGAP011771 as a candidate for *Anopheles* pst. We designed a double-stranded RNA construct to silence the gene, and measured viral loads by the duplex Taqman RT-qPCR assay (silencing efficiency shown in Supplementary Fig. S7). A non-significant increase of AnCPV-infected mosquitoes was observed after depletion of pst candidate AGAP011771 in four biological replicates (Supplementary Fig. S7a).

## Discussion

The *Anopheles* virome is comprised of a number of viruses, among them insect specific viruses (ISVs)^5^. These ISVs are maintained by vertical transmission but their biology in *Anopheles* has not been examined. The goal of this study was to characterize the biological interaction of two ISVs, *Anopheles* C virus (AnCV) and *Anopheles* cypovirus (AnCPV), with *An*. *coluzzii*, the mosquito host in which they were discovered by deep sequencing of RNA and *de novo* assembly^16^. Both viruses have also both been identified in wild-caught *Anopheles* of various species.

Here, we studied the interaction and immune response to AnCV and AnCPV in co-infected *An*. *coluzzii*. In order to quantify the two viruses simultaneously, we developed a duplex Taqman real-time RT-PCR assay that is specific, sensitive, and rapid. We detected a consistent pattern of virus abundance corresponding to stages of host development. We showed that AnCV uses a transovarial, intraembryonic route of transmission. The mechanism of AnCPV persistence is less clear because we did not detect transovarial transmission. However, AnCPV is present at lower levels in the larval stage than AnCPV, and therefore the transmission experiment may have had less sensitivity to detect AnCPV. It was not possible to test adults emerging from the single-egg hatching experiment because larvae grown from eggs hatched in individual wells did not efficiently develop to adults. Both viruses are present in oviposition fluid and NaOCl-untreated eggs of *An*. *coluzzii*, but the virus-positive oviposition fluid did not infect *An*. *stephensi* larvae by feeding in larval water. Under the same conditions, we previously demonstrated that filtered infected total larval extract infected *An*. *stephensi* larvae. The difference could be explained if the viral particles in the oviposition fluid have low environmental stability and/or abundance. Taken together, transovarial transmission was demonstrated for AnCV, and this also appears to be the most likely explanation for AnCPV persistence, but the latter will require further work to detect. Transovarial transmission was demonstrated for DENV and Zika virus in *Aedes* mosquitoes^37,38^, but data are lacking on transmission modes of ONNV in *Anopheles*.

We identified a significant dependence of the abundance distributions of the two viruses in individual mosquitoes. The inversely correlated abundance profiles could be a consequence of cellular interference, manipulation of host immunity, or segregating genetic variation in the host. In order to survey potential host immune effects, we queried the most important immune signaling pathways for influence on the two virome members. We identified a role of the Toll pathway in limiting AnCPV abundance. The effect was only detectable in the background of low infection with AnCV. One hypothesis could be that AnCV inhibits Toll pathway activation, which was previously shown for ONNV in *Anopheles*^12^. However, in the case of ONNV, adventitious stimulation of Toll displayed a deleterious effect on ONNV replication, while in the current study stimulation of Toll by depletion of Cactus did not influence AnCV levels. It thus appears more likely that AnCV may express an inhibitor of an unknown Toll-dependent antiviral effector, and not an inhibitor of Toll pathway activation. Hypothetically, AnCV could inhibit expression of the antiviral factor gene, or could directly inhibit the activity of the antiviral effector. Other dicistroviruses such as Drosophila C virus and Cricket paralysis virus express inhibitors of different targets in the RNAi pathway^39^. Further work could include assaying expression of Toll-response genes in the presence and absence of AnCV infection, in order to test the first hypothesis that AnCV inhibits antiviral gene expression.

We also identified a role of the JAK/STAT pathway in promoting infection of *An*. *coluzzii* by AnCPV. Silencing of the JAK/STAT positive regulator STAT-A to inhibit pathway activity reduces AnCPV infection prevalence. JAK/STAT is involved in maintaining homeostasis of the bacterial microbiome, and this result might suggest an implication of the bacterial microbiome in regulating virome composition. In contrast to the positive effect of JAK/STAT activity on AnCPV levels, the same pathway is antagonistic to ONNV during the primary midgut infection in *An*. *coluzzii*^12^, and has no effect on the disseminated systemic ONNV infection after intrathoracic injection^14^. In *Drosophila*, the loss of *hop*, which regulates the single STAT factor, STAT92E increases the viral loads of *Drosophila* C virus and elevates host mortality^29^. In contrast, depletion of STAT-A in *An*. *gambiae* reduces the number of *Plasmodium* oocysts in the midgut but increases their proportional survival^40^. These results observed for different infections indicate a diversity of effects of the JAK/STAT pathway in *Anopheles* depending on pathogen type and other factors.

Finally, we identified and functionally assayed a candidate for *Anopheles* pastrel. Mosquitoes depleted of transcript for this candidate displayed a non-significant tendency to increased infection by AnCPV. In order to further characterize the pastrel candidate, as well as a weak potential effect of the Imd pathway, it may be necessary to eliminate or control for the effects of the coinfection of AnCV and AnCPV, which are clearly not independent.

Work still remaining includes examining the influence of the virome upon malaria and ONNV susceptibility and transmission. Our initial attempts to test the influence of the two viruses upon *P*. *falciparum* infection were inconclusive, because the persistent infection of *An*. *coluzzii* colonies with negatively correlated loads of the two viruses is a confounding factor that requires large sample sizes for statistical power of correlation. *An*. *coluzzii* lines carrying only one of AnCV and/or AnCPV would permit controlled reinfection studies. There are interesting examples from other mosquitoes of virome interaction with transmissible pathogens. The insect specific flavivirus of *Aedes*, Cell fusing agent virus, enhances DENV replication and the reciprocal in *Aedes* cells^41^. Palm Creek virus, another insect specific flavivirus, causes reduced replication of the West Nile virus and Murray Valley encephalitis arboviruses in *Aedes* cells^42^. However, these studies were done in cultured cells, and studies on interactions between ISVs and transmissible pathogens such as arboviruses and *Plasmodium in vivo* in mosquitoes, closer to the natural biological conditions, remain to be done.

In the current work, we showed that *An*. *coluzzii*, one of main human malaria vectors in Africa, uses classical signaling pathways of immune response to control at least one ISV of the natural virome, AnCPV. The other ISV carried by this mosquito colony, AnCV, appears to be more difficult for the host to control, because its replication was not limited by the pathways or effectors tested. We speculate that AnCV may inhibit an effector of the Toll pathway, an antiviral effector that in turn reduces AnCPV replication when induced. However, *Anopheles* antiviral mechanisms against arbovirus infection may be quite efficient, based on the relative lack of arbovirus transmission by *Anopheles* despite highly anthropophilic feeding behavior, including on viremic hosts. Nevertheless, ONNV transmission is the exception that indicates arbovirus transmission by *Anopheles* is possible, so it is a biological puzzle that transmission is apparently restricted to just one virus.

Identifying the community of natural viruses inhabiting the *Anopheles* host niche will help clarify the biology underlying the apparent inefficiency of arbovirus transmission by *Anopheles*, and may suggest new tools to raise the barrier to arbovirus transmission by the more efficient *Aedes* and *Culex* vectors. The current study shows that *Anopheles* ISVs, because of their non-pathogenicity to humans, can be used as simple and tractable models to dissect the diversity of interactions between *Anopheles* mosquitoes and RNA viruses.

## Methods

### Mosquitoes

The *Anopheles coluzzii* Ngousso strain was initiated in Cameroon in 2006^43^ and was obtained by the Institut Pasteur CEPIA facility in 2008. The *Anopheles stephensi* strain SDA500 was initiated in Pakistan in 1982^44^ and was obtained by the Institut Pasteur CEPIA facility between 2000–2004. Larvae were grown in distilled water supplemented with 0.01% of mineral salt, and fed on Friskies cat food. Adults were reared at 28 °C ±1, at 80% ±5 humidity on a 12 h light–dark cycle. Adults were fed on sterile filtered and autoclaved 10% sucrose solution and females were blood-fed on anaesthetized rabbits for colony maintenance.

### Molecular detection and quantification of virus RNA

Template RNA was extracted from individual or pooled mosquitoes using Direct-zol RNA MiniPrep reagents with DNase I treatment (Zymo Research). cDNA was synthesized from the RNA using Moloney Murine Leukemia Virus Reverse Transcriptase (Invitrogen) and random hexamer primers (Roche).

Virus detection of AnCV and AnCPV by simple RT-PCR followed by agarose gel analysis was carried out as described^16^. A duplex Taqman real-time RT-PCR assay (Taqman RT-qPCR) was developed (Supplementary Table S2) to detect and quantify the two viruses simultaneously. Taqman primers and probes were designed using Primer Express Software v2.0 (Applied Biosystems). Probe and primer sequences are listed in Supplementary Table S3. All have a minor groove binder/non-fluorescent quencher (MGB-NFQ) at the 3′ end and the 6-carboxyfluorescein (6-FAM), VIC and NED fluorophores at the 5′ end for AnCV, AnCPV and The *An*. *coluzzii* rpS7 gene respectively. Taqman RT-qPCR was performed with Taqman Universal Master Mix II, with UNG on a QuantStudio 12 K Flex instrument (Applied Biosystems). Primer concentration was 0.9 μM and probe concentration 0.25 μM. The cycling protocol was a hold step 50 °C for 2 min and 95 °C for 10 min, followed by the PCR stage of 40 cycles: 95 °C for 15 secs and 60 °C for 1 min. Negative controls were run with each test. A pool of 10 mosquitoes harboring both viruses was used to make the standard curve. Ten-fold serial dilutions were made between the five dilutions (dil1 to dil5) of the standard curve to test efficiency, specificity and sensitivity. All reactions were performed in triplicate.

No amplification was observed in negative controls with no template. Sensitivity was approved with amplification in the five dilutions of the standard sample (3000 ng for dil1 to 0.03 ng for dil5), and Ct values were inversely proportional to the expected amount of target nucleic acid in each dilution. Efficiency was over 90% in all assays. The *An*. *coluzzii* rpS7 gene was amplified in the same plate as the endogenous control gene for relative quantification.

### Egg treatment to determine virus transmission route

Eggs were treated with sodium hypochlorite (NaOCl) to eliminate viruses on the egg surface. In a previous study, eggs of the beet armyworm (*Spodoptera exigua***)** were decontaminated of nucleopolyhedroviruses (genus *Alphabaculovirus*, family *Baculoviridae*) by treatment with 0.25ppm (0.000025%) NaOCl^45^. We determined that *Anopheles* eggs were more resistant to NaOCl, possibly because of the impermeable egg chorion, and that eggs treated with 0.025% were still viable. Consequently, 0.025% NaOCl was used in the current study.

A batch of freshly laid *An*. *coluzzii* Ngousso strain eggs was divided into 3 batches. The experimental batch was treated with 0.025% NaOCl for 10 min, rinsed with distilled water, and placed as individual eggs in a well of a 24-well plastic culture plate for hatching and growth until L3/L4 larval stages. The control batch was mock-treated by only rinsing with distilled water (with no NaOCl treatment) and placed as individual eggs in a well of a 24-well plastic culture plate for hatching and growth until L3/L4 larval stages. L3/L4 larval stages from each batch were collected and tested using an RT-PCR assay for presence and abundance of AnCV and AnCPV virus genomes. The experiment was carried out in two replicates.

An additional egg batch was washed with 300 µl of distilled water, which was then collected and filtered with the 5 µm and 0.2 µm filters to remove bacteria. The filtered solution containing oviposition fluid was tested using the duplex Taqman RT-qPCR assay for presence and abundance of AnCV and AnCPV virus genomes. The liquid rinsed from the surface of eggs containing oviposition fluid was fed in the larval water to L2 larvae of *An*. *stephensi*. The experiment was carried out in two replicates.

### Gene silencing assays

Gene-specific fragments of Ago2, Cactus, the pastrel candidate AGAP011771, Rel2, STAT-A, and GFP control were generated by PCR using primers tagged at their 5′ end with T7 promoter sequences. All T7 primer sequences are listed in Supplementary Table S3. The PCR products were used as template for *in vitro* dsRNAs synthesis using the MEGAscript RNAi Kit (Ambion). The targeted gene was silenced by injecting 500 ng of dsRNA into the thorax of ice-anesthetized 1–2 days old post-emergence of *An*. *coluzzii* females using a nanoinjector (Nanoject II; Drummond Scientific) and glass capillary needle as previously described^46^. Four days after the dsRNA treatment, silencing of the target gene was verified using total RNA from a pool of five mosquitoes by SYBR Green RT-qPCR for Ago2, Rel2 and pastrel, while RT-PCR followed by detection on agarose gels was used for Cactus and STAT-A. RNA was extracted using Direct-zol RNA MiniPrep reagents with DNase I treatment (Zymo Research). cDNA synthesis was done using Moloney Murine Leukemia Virus Reverse Transcriptase (Invitrogen) and random hexamer primer (Roche). The RT-qPCR primers used for gene expression are given in the Supplementary Table S3.

Total RNA extracted from individual mosquitoes 4 days post-dsRNA injection was used to assess virus infection intensity and prevalence by duplex Taqman real-time RT-PCR assay using relative quantification. The rpS7 gene was used as a housekeeping calibrator for normalization of nucleic acid quantity. Analysis of the expression of transcript relative to rpS7 was performed according to the 2^−ΔΔCt^ method^47^. Intensity is the log10 of relative expression of virus to rpS7.

### Statistical analysis

All statistical details are presented in Supplementary Table S4. For comparisons of virus infection prevalence of different mosquito developmental stages, the chi-square test was applied, and p-values with a null distribution were estimated by the Monte-Carlo method with 10,000 permutations. Multiple testing correction was done by the Bonferroni method. Box and bar plots were made using the beeswarm package in R^48^. For viral load (infection intensity), statistical significance was determined using the Wilcoxon–Mann–Whitney rank-sum test, and p-values were assessed with a null distribution of the statistical test approximated using Monte-Carlo resampling with 1,000,000 permutations. The p-values from independent tests of significance were combined using the meta-analytical approach of Fisher^49^.

## Supplementary information

Supplementary Information

## Data Availability

All data are available in the article.
