# Supplementary material for: Interaction of RNA viruses of the natural virome with the African malaria vector, Anopheles coluzzii
Source: Sci Rep. 2019 Apr 19;9:6319. doi: 10.1038/s41598-019-42825-3 (PMC6474895; doi:10.1038/s41598-019-42825-3)
Supplement: Supplementary file 1 — Supplementary Information [file 41598_2019_42825_MOESM1_ESM.docx]

**Interaction of RNA viruses of the natural virome with the African malaria vector, *Anopheles coluzzii***

Ferdinand Nanfack-Minkeu ^1,2,3^, Christian Mitri ^1,2^, Emmanuel Bischoff ^1,2^, Eugeni Belda ^1,2^, Isabelle Casademont ^2,4^, Kenneth D Vernick ^1,2,*^

^1^ Department of Parasites and Insect Vectors, Unit of Genetics and Genomics of Insect Vectors, Institut Pasteur, Paris 75724 CEDEX 15, France.

^2^ CNRS, Unit of Evolutionary Genomics, Modeling and Health (UMR2000), 28 rue du Docteur Roux, 75015 Paris, France.

^3^ Sorbonne Université, Graduate School of Life Sciences ED515, UPMC - Université Pierre et Marie Curie - Paris 6, 4 Place Jussieu, 75252 Paris, France.

^4^ Unit of Functional Genetics of Infectious Diseases, Department Genome and Genetics, Paris, France.

* Correspondence:

email: kvernick@pasteur.fr

address: Institut Pasteur, GGIV, 28 rue du Dr Roux, 75724 Paris Cedex 15, FRANCE

**Supplementary Figure Legends**

**Supplementary Figure S1. Absence of detectable DNA forms of viral genomes of AnCV and AnCPV in An. coluzzii Ngousso strain.** PCR primers targeted the RNA-dependent RNA polymerase genes of each virus. DNA template was extracted from pools of 5 to 10 mosquitoes each using DNAzol, and cDNA was generated from RNA of pools 5 to 10 mosquitoes each using Trizol. PCR reactions of DNA and cDNA templates were run simultaneously. PCR of the ribosomal protein S7 spanned an intron, so that product sizes confirm the difference between smaller spliced cDNA and larger unspliced DNA template. Water was used as negative control. The amplification of AnCV and AnCPV was obtained only from cDNA, indicating the absence of DNA forms of these RNA viruses. The uncropped gel image is shown below the figure.

**Supplementary Figure S2. Individual replicate tests and p-values of virus abundance correlation.** **A.** Two replicates for Figure 3a. **B.** Five replicates for Figure 3b.

**Supplementary Figure S3. Effect of Toll activation on the aggregate mosquito sample.** **A.** Toll activation produces a non-significant tendency towards lower infection intensity by AnCV in 3 independent replicates. dsCactus indicates mosquitoes treated with dsCactus and dsGFP is the control group injected with irrelevant dsRNA. Silencing efficiency was verified by RT-PCR (Supplementary Figure S3c). x-axis, dsRNA treatment, y-axis represents AnCV expression relative to ribosomal protein S7 in log10 scale. Red, highly infected mosquitoes for indicated virus (log10 of relative expression > 0). Green, low infected mosquitoes for indicated virus (log10 of relative expression < 0). (n) indicates the total number of mosquitoes dissected for each of the biological replicates. Statistical differences were first tested independently within replicates with the Wilcoxon-Mann-Whitney test using 100,000 permutations for assessing the p-value. Then if individual replicates showed consistent direction of change, individual p-values were combined using the meta-analytical approach of Fisher. **B.** Toll activation produces inconsistent effects on AnCPV infection intensity. Details as in part A. **C.** Validation of Cactus silencing after injection of dsCactus, three independent replicates detected by RT-PCR followed by agarose gel. Replicate 1, R1, replicate 2, R2, replicate 3, R3. The uncropped gel images are shown below the figure.

**Supplementary Figure S4. Validation of STAT-A silencing.** Two independent replicates, detected by RT-PCR followed by agarose gel. Replicate 1, R1, replicate 2, R2. The uncropped gel images are shown below the figure.

**Supplementary Figure S5. RNA interference activity does not significantly influence AnCV and AnCPV infection. A.** Details as in Supplementary Figure S3a legend, except DsAgo2 indicates the mosquitoes injected with double stranded RNA directed against Ago2. **B.** Validation of Ago2 silencing. Two independent replicates, detected by SYBR Green RT-qPCR. Fold-change indicates Ago2 expression after normalization with the ribosomal protein S7 gene, taking expression in dsGFP controls as 100%. Ago2 expression was reduced by at least 60% in replicate 1 and at least 40% in replicate 2.

**Supplementary Figure S6. Imd pathway activity displays a non-significant positive effect for AnCPV infection. A.** Details as in Supplementary Figure S3a legend, except DsRel2 indicates the mosquitoes injected with double stranded RNA directed against Rel2. Statistical differences were first tested independently within replicates with the Wilcoxon-Mann-Whitney test using 100,000 permutations for assessing the p-value (given above each plot). Then since individual replicates showed consistent direction of change, individual p-values were combined using the meta-analytical approach of Fisher (combined p-value=0.326, chi-square=6.94, df=6,). **B.** Validation of Rel2 silencing. Details as in Supplementary Figure S5b legend. Rel2 expression was reduced by at least 60% in replicates 1 and 2 and 35% in replicate 3.

**Supplementary Figure S7. Candidate pastrel ortholog PEST AGAP011771 displays a non-significant protective tendency against AnCPV infection. A.** Details as in Supplementary Figure S3a legend, except DsPst indicates the mosquitoes injected with double stranded RNA directed against pst candidate, AGAP011771. **B.** Validation of pst candidate AGAP011771 silencing. Details as in Supplementary Figure S5b legend. Rel2 expression was reduced by at least 60% in replicates 2, 3 and 4 and at least 40% in replicate 1.

**Supplementary Figure S1**

Uncropped gel:


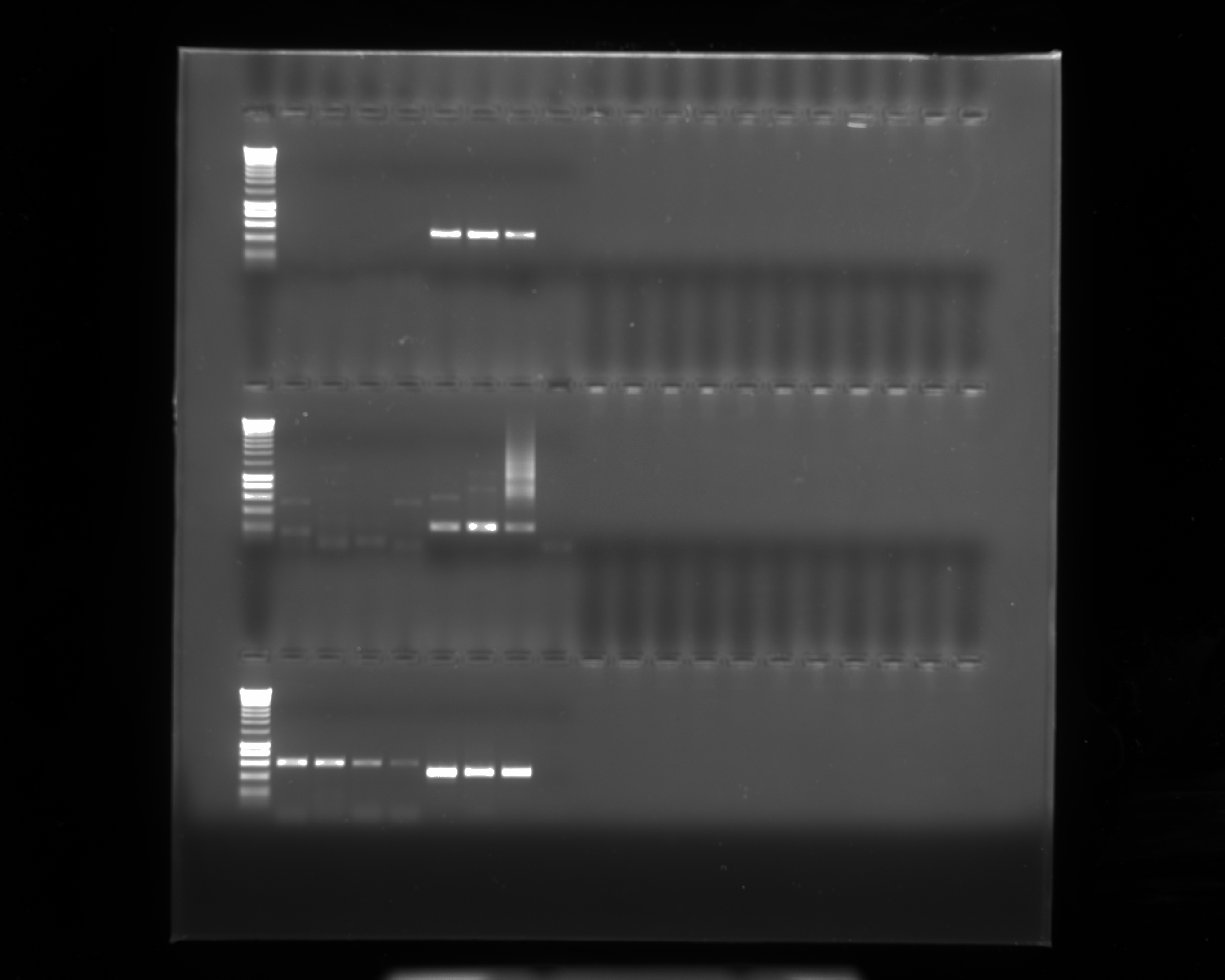


**Supplementary Figure S2a**

**Supplementary Figure S2b**

**Supplementary Figure S3a**

**Supplementary Figure S3b**

**Supplementary Figure S3c**

**Supplementary Figure S4**

**Supplementary Figure S5a**

**Supplementary Figure S5b**

**Supplementary Figure S6a**

**Supplementary Figure S6b**

**Supplementary Figure S7a**


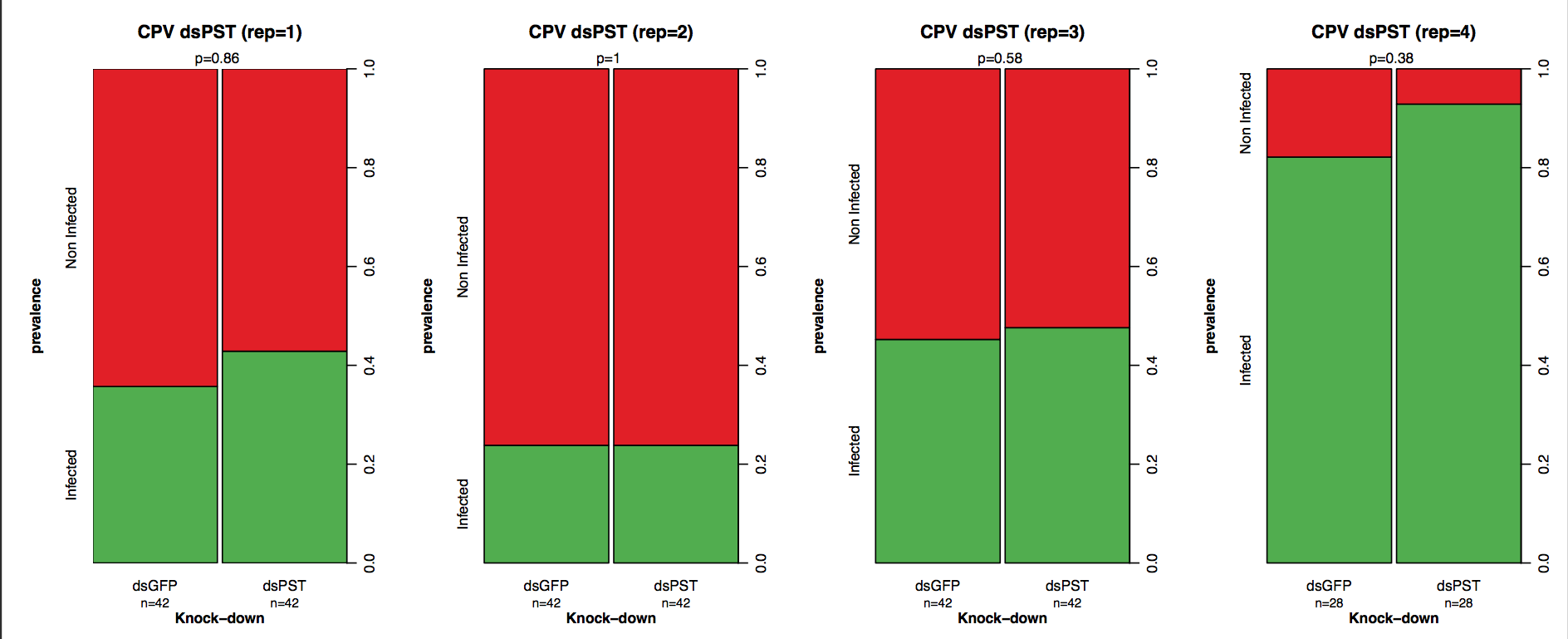


**Supplementary Figure S7b**

**Supplementary Table S1. Variation of infection prevalence in *An. coluzzii* developmental stages for Anopheles C Virus (AnCV) and Anopheles Cypovirus (AnCPV).**

Average prevalence for the 2 biological replicates and 95% confidence interval are given for each virus and developmental stage.

|  |  | | | | |
| --- | --- | --- | --- | --- | --- |
| Viruses | Larvae (L3/L4) | Pupae | Imagos | Adults, 1 week | Adults, 2 weeks |
| AnCV | 93.33  [80.27 -106.40] | 89.65  [69.38-109.93] | 35.90  [21.86 - 49.94] | 41.11  [33.26 - 48.96] | 34.21  [12.87 - 55.54] |
| AnCPV | 7.78  [2.02 - 13.54] | 11.19  [6.98 - 15.39] | 6.74  [2.97 - 10.52] | 41.11  [19.01- 63.21] | 25.95  [17.17 - 34.74] |

**Supplementary Table S2. Summary of settings for duplex Taqman real-time RT-PCR assay (Taqman RT-qPCR).**

Efficiency (Eff) = 10^-(1/slope)^ -1, R, reproducibility, Dil 1, dilution 1 of standard sample.

| **Assays** | **R^2^** | **Slope** | **Eff (%)** | **Ct Mean**  **Dil1** | **Ct Mean** |
| --- | --- | --- | --- | --- | --- |
| AnCV simplex | 0.996 | -3.431 | 95.63 | 12.169 | 27.339 |
| AnCV duplex | 0.998 | -3.352 | 98.78 | 13.008 | 28.657 |
| AnCPV simplex | 0.989 | -3.313 | 100.40 | 19.663 | 19.095 |
| AnCPV duplex | 0.997 | -3.186 | 105.99 | 20.709 | 18.924 |

**Supplementary Table S3. List of primers and probe sequences.** F, forward and R, reverse, q, quantitative, taq, Taqman.

| Virus/Genes | Primers | Sequences |
| --- | --- | --- |
| RT-PCR | | |
| AnCV | AnCV-F | CAAGGAGGCTTTTTGAGTGC |
|  | AnCV-R | GCTTTGGGTAAGCTGTCGAG |
| AnCPV | AnCPV-F | TGAGCGAATCGTGCACCATG |
|  | AnCPV-R | GGTTTTCCGACTAGCCTTCC |
| rpS7 | S7-F | AGGCGATCATCATCTACGTGC |
|  | S7-R | GTAGCTGCTGCAAACTTCGG |
| RT-qPCR SYBR Green | | |
| rpS7 | S7-qF | AGAACCAGCAGACCACCATC |
|  | S7-qR | GCTGCAAACTTCGGCTATTC |
| AnCV | AnCV-qF | ATCGCGTAATTAGGGCTCCA |
|  | AnCV-qR | TTGAGACACAGGACAGCGAT |
| AnCPV | AnCPV-qF | TCGACAGATGCAGCTCAAGG |
|  | AnCPV-qR | AACCACGCGTCACTTCAAGA |
| qPCR Taqman | | |
| AnCV | AnCV-Taq-F | ACCAGGAAAGAACGACGTAGACA |
|  | AnCV-Taq-R | CGCACCCTTAACAGCTTTGG |
|  | AnCV-Probe (FAM) | TTTCATGCGCAGGCTCGACAGC |
| AnCPV | AnCPV-Taq-F | CAGCTCAAGGTAAACAGGTTGGT |
|  | AnCPV-Taq-R | CGTCTGAGTTGTCGCGAATAAC |
|  | AnCPV-Probe (VIC) | ATTCAATGGACCTTCAAGAC |
| rpS7 | Ag-S7- Taq-F | CAAGCGTATCCGCGTCAAG |
|  | Ag-S7- Taq-R | GGTGGTCTGCTGGTTCTTATCC |
|  | Ag-S7- Probe (NED) | TCGACGGATCCCAGCTGATAAAGGTG |
| RT-PCR : double stranded RNA synthesis | | |
| GFP | T7-GFP-F | GAATTGTAATACGACTCACTATAGGGCATGGTGAGCAAGGGCGAG |
|  | T7-GFP-R | GAATTGTAATACGACTCACTATAGGGCTTACTTGTACAGCTCGTC |
| AGO2 | T7-Ago2-F | TAATACGACTCACTATAGGGGTTCGCGCCCATACCTAAA |
|  | T7-Ago2-R | TAATACGACTCACTATAGGTTGTTTTTGTTCAGCGCCTG |
| Cactus | T7-Cactus-F | TAATACGACTCACTATAGGTGGTGCGTCGATTGCTGG |
|  | T7-Cactus-R | TAATACGACTCACTATAGGCTTTCGTTCAAGTTCTGTGC |
| REL2 | T7-REL2-F | TAATACGACTCACTATAGGGCAACAGCAGCAACAACATC |
|  | T7-REL2-R | TAATACGACTCACTATAGGGCACAGGCACACCTGATTGAG |
| STAT A | T7-STATA-F | TAATACGACTCACTATAGGCCGGAGAGCAACTTCACGAT |
|  | T7-STATA-R | TAATACGACTCACTATAGGGATGAACGTGTTGTAATGAGC |
| AGAP011771 (Pastrel) | T7-AGAP011771-F | TAATACGACTCACTATAGGACGAGGATGATGGTAGGCTG |
|  | T7-AGAP011771-R | TAATACGACTCACTATAGG AGTAATTCGGATCCTGGCGG |
| RT-qPCR SYBR Green to verify gene expression after silencing | | |
| Ago2 | Ago2-qF | CCAAGCCGACCAAGTACG |
|  | Ago2-qR | GCAAACAGGTGGCACAGATT |
| AGAP011771 (pastrel) | AGAP011771-qF | GCAACAACAAGTACCCGTCG |
|  | AGAP011771-qR | TACCGCCCGACAGAAAGATG |
| rpS7 | S7-qF | AGAACCAGCAGACCACCATC |
|  | S7-qR | GCTGCAAACTTCGGCTATTC |
| REL2 | REL2-qF | CTCAATCAGGTGTGCCTGTGC |
|  | REL2-qR | GGTGGTGCTGAGCCGGCAGATC |

**Supplementary Table S4. Detailed statistical test results.** Cond, condition. #, number. WMW, Wilcoxon Mann Whitney test, ds, double stranded.

|  |  | **Figure 3. Inverse correlation between AnCV and AnCPV infection levels in An. coluzzii.** | | | | | |  |  |  |  |
| --- | --- | --- | --- | --- | --- | --- | --- | --- | --- | --- | --- |
|  | samples |  |  | WMW test for each replicate |  |  |  | Fisher's combined probability |  |  |  |
|  | **cond** | **replicate** | **# samples** | **chi2** | **p-value** |  |  | **Chi-2** | **df** | **p-value** | **Viruses** |
| **Fig. 3a** | Non injected | 1 | 30 | 30 | 7,00E-05 |  |  | 26.1 | 4 | 3e-05 | ACV/CPV |
| **Fig. 3a** | Non injected | 2 | 30 | 16.6 | 0.0303 |  |  | — | — | — | ACV/CPV |
| **Fig.3b** | dsGFP | 1 | 28 | 17.3 | 4,00E-05 |  |  | 78.4 | 10 | 1e-12 | ACV/CPV |
| **Fig.3b** | dsGFP | 2 | 28 | 9.02 | 0.00842 |  |  | — | — | — | ACV/CPV |
| **Fig.3b** | dsGFP | 3 | 28 | 17.3 | 5,00E-05 |  |  | — | — | — | ACV/CPV |
| **Fig.3b** | dsGFP | 4 | 28 | 6.6 | 0.0569 |  |  | — | — | — | ACV/CPV |
| **Fig.3b** | dsGFP | 5 | 63 | 31.7 | 1,00E-05 |  |  | — | — | — | ACV/CPV |
|  |  | **Supplementary Figure S3a. Supplementary Figure S3. Effect of Toll activation on the aggregate mosquito sample** | | | | | |  |  |  |  |
|  |  |  |  |  |  |  |  |  |  |  |  |
| **Fig. S3a** | dsGFP/dsCactus | 1 | 28 |  | 0.06204 |  |  | 8.42 | 6 | 0.209 | ACV |
| **Fig. S3a** | dsGFP/dsCactus | 2 | 28 |  | 0.82829 |  |  |  |  |  | ACV |
| **Fig. S3a** | dsGFP/dsCactus | 3 | 63 |  | 0.28872 |  |  |  |  |  | ACV |
|  |  | **Supplementary Figure S3b. Supplementary Figure S3. Effect of Toll activation on the aggregate mosquito sample** | | | | | | | |  |  |
| **Fig. S3b** | dsGFP/dsCactus | 1 | 28 |  | 0.9818 |  |  | NA | NA | NA | CPV |
| **Fig. S3b** | dsGFP/dsCactus | 2 | 28 |  | 0.0378 |  |  | — | — | — | CPV |
| **Fig. S3b** | dsGFP/dsCactus | 3 | 63 |  | 0.1738 |  |  | — | — | — | CPV |
|  |  | **Figure 4. Toll pathway activity limits levels of AnCPV in An. coluzzii.** | | | | |  |  |  |  |  |
| **Fig.4** | dsGFP | 1 | 20 |  | 0.005 |  |  | 17.5 | 6 | 0.00753 | CPV |
| **Fig.4** | dsGFP | 2 | 19 |  | 1 |  |  | — | — | — | CPV |
| **Fig.4** | dsGFP | 3 | 41 |  | 0.03 |  |  | — | — | — | CPV |
| **Fig.4** | dsCactus | 1 | 25 |  | 0.005 |  |  | — | — | — | CPV |
| **Fig.4** | dsCactus | 2 | 19 |  | 1 |  |  | — | — | — | CPV |
| **Fig.4** | dsCactus | 3 | 40 |  | 0.03 |  |  | — | — | — | CPV |
|  |  | **Figure 5. JAK/STAT pathway activity promotes AnCPV infection in An. coluzzii.** | | | | | |  |  |  |  |
| **Fig.5** | dsGFP/dsSTAT_A | 1 | 42 | 0.933 | 0.467 |  |  | 11.4 | 4 | 0,0228 | CPV |
| **Fig.5** | dsGFP/dsCactus | 2 | 42 | 8.4 | 0.00732 |  |  | — | — | — | CPV |
|  |  | **Supplementary Figure S5. RNA interference activity does not significantly influence AnCV and AnCPV infection.** | | | | | | | |  |  |
| **Fig.S5** | dsGFP/dsAgo2 | 1 | 28 |  | 0.58845 |  |  | NA | NA | NA | ACV |
| **Fig.S5** | dsGFP/dsAgo2 | 2 | 28 |  | 0.0834 |  |  | — | — | — | ACV |
| **Fig.S5** | dsGFP/dsAgo2 | 1 | 28 |  | 0.37776 |  |  | — | — | — | CPV |
| **Fig.S5** | dsGFP/dsAgo2 | 2 | 28 |  | 0.22433 |  |  | — | — | — | CPV |
|  |  | **Supplementary Figure S6. Imd pathway activity displays a non-significant positive effect for AnCPV infection.** | | | | | | | |  |  |
| **Fig.S6** | dsGFP/dsREL2 | 1 | 42 | 1.42 | 0.341 |  |  | 6.94 | 6 | 0.326 | CPV |
| **Fig.S6** | dsGFP/dsREL3 | 2 | 42 | 2.55 | 0.182 |  |  | — | — | — | CPV |
| **Fig.S6** | dsGFP/dsREL4 | 3 | 42 | 1.02 | 0.501 |  |  | — | — | — | CPV |
|  |  | **Supplementary Figure S7. Candidate pastrel ortholog PEST AGAP011771 displays a non-significant protective tendency against AnCPV infection.** | | | | | | | | |  |
| **Fig.S7** | dsGFP | 1 | 15 | 0.0379 | 1 |  |  | 3.32 | 8 | 0.913 | CPV |
| **Fig.S7** | dsGFP | 2 | 10 | 0 | 1 |  |  | — | — | — | CPV |
| **Fig.S7** | dsGFP | 3 | 19 | 1.24 | 0.333 |  |  | — | — | — | CPV |
| **Fig.S7** | dsGFP | 4 | 23 | 0.477 | 0.572 |  |  | — | — | — | CPV |
| **Fig.S7** | dsPST | 1 | 18 | 0.0379 | 1 |  |  | — | — | — | CPV |
| **Fig.S7** | dsPST | 2 | 10 | 0 | 1 |  |  | — | — | — | CPV |
| **Fig.S7** | dsPST | 3 | 20 | 1.24 | 0.333 |  |  | — | — | — | CPV |
| **Fig.S7** | dsPST | 4 | 26 | 0.477 | 0.572 |  |  | — | — | — | CPV |
